# An evaluation of the use and efficacy of behavioural therapy when treating paediatric patients with radiation therapy

**DOI:** 10.1002/jmrs.705

**Published:** 2023-07-13

**Authors:** Brianna McCoola, Julie‐Anne Outhwaite, Marika Lathouras, Anita Pelecanos, Jemma Blyth, Amanda Carter, Yvonne Hastings, Greg Rattray, Robyn Cheuk

**Affiliations:** ^1^ Department of Radiation Therapy, Cancer Care Services, Royal Brisbane and Women's Hospital Metro North Hospital and Health Service Brisbane Queensland Australia; ^2^ Statistics Unit QIMR Berghofer Medical Research Institute Brisbane Queensland Australia; ^3^ Queensland Children's Hospital Children's Health Queensland Brisbane Queensland Australia

**Keywords:** anaesthetic, paediatric, play, radiotherapy, therapy

## Abstract

**Introduction:**

The paediatric radiation therapy group (PRTG) provided a multidisciplinary network to support patients accessing radiation therapy (RT). This study aims to evaluate the use and efficacy of behaviour therapy practices used by the PRTG.

**Methods:**

A retrospective cross‐sectional review of general anaesthetic (GA) utilisation for paediatric patients was completed between 1 January 2010 and 30 June 2014. The PRTG incorporated behavioural therapy techniques into all appointments but offered additional play appointments to children unable to comply with the requirements of RT. This aimed to increase their compliance and minimise GA use.

**Results:**

Two‐hundred and seventy‐four patients had 5402 occasions of service, of which 1361 were delivered under GA (25.2%). Two‐hundred and fifty‐seven patients met the eligibility criteria. Patients under 8 years who required GA for their entire treatment reduced for each year of increase in age (odds ratio 0.37, 95% confidence interval 0.27–0.51, *P* < 0.001). Participants 3 years and younger were shown not as likely to change their GA requirements with the use of play appointments. Seventy‐eight per cent (83/106) of 3–8‐year‐olds used no GA or ceased GA during treatment.

**Conclusions:**

Most paediatric patients <3 years will gain minimal benefit to reduce GA use from additional play appointments. Children older than nine were not likely to require play appointments to be compliant with RT. Encouragingly, 53.3% of 3–8‐year‐olds who were categorised as full GA after CT planning did not continue to a full course of GA due to the behavioural therapy interventions of the PRTG.

## Graphical abstract

The application of behavioural therapy practices for paediatric patients in radiation therapy (RT) has been reviewed against general anaesthetic use to determine the efficacy of the approach and identify trends at the Royal Brisbane & Women's Hospital, Australia. It was found that paediatric patients younger than 3 years and older than 9 years were not likely to benefit or require additional behavioural therapy interventions to be compliant with the requirements of RT.

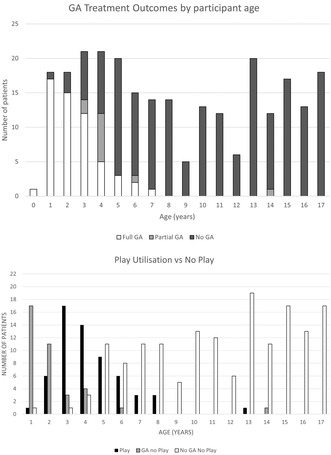

## Introduction

Radiation therapy (RT) has long been an important treatment approach for paediatric oncology patients. To ensure optimal RT outcomes, minimal to no motion during treatment is crucial and therefore, patient compliance is essential.^1^ This can be challenging for paediatric patients due to their levels of cognitive development and their ability to feel comfortable and safe in a hospital environment related to many factors over which clinical staff have little control.^2^, ^3^, ^4^, ^5^, ^6^, ^7^, ^8^, ^9^ To overcome non‐compliance and ensure RT treatment accuracy, many healthcare centres sedate young children daily for RT procedures.^2^, ^3^, ^4^, ^5^, ^6^, ^7^, ^8^, ^9^ Daily sedation for a child may increase the risk of medical complications, affecting nutrition and overall health and well‐being.^6^, ^9^ Anaesthesia also comes at a high financial cost to the healthcare facility.^2^, ^7^, ^9^

Behavioural therapy techniques have been recognised as a valuable strategy to reduce paediatric distress and enhance compliance within healthcare.^2^, ^7^ Radiation therapists (RTs) and Certified Child Life specialists (CCLS) incorporate techniques of desensitisation, behavioural rehearsal, cognitive distraction and positive reinforcement into paediatric oncology care.^2^, ^7^, ^9^, ^10^, ^11^, ^12^, ^13^, ^14^, ^15^, ^16^, ^17^, ^18^ While published literature on general anaesthetic (GA) use in children during RT is minimal, papers published concluded that behavioural therapy interventions can reduce anaesthesia use.^9^, ^16^, ^18^ In the US, Scott^16^ published an 8‐year retrospective analysis comparing GA use before and after CCLS integration into the model of care, showing a reduction in GA use of 16.2% in 3–12‐year‐olds. This study also quantified the financial benefit to their department in employing CCLS, with the estimated cost‐saving approaching $8000 US per child. Ntoukas et al^18^ reported an 8‐year study showing that when paediatric nurses worked with RTs to implement behavioural therapy practices, GA was only required in 12.8% of occasions of service in under 19‐year‐olds.

Despite this evidence base, there has been a paucity of research conducted in the context of the Australian healthcare environment. While strong anecdotal support exists, there is little empirical evidence for the use of behavioural models of care in the treatment of paediatric oncology patients.

Cancer Care Services RT department at the Royal Brisbane and Woman's Hospital (RBWH), previously known as the Queensland Radium Institute (QRI) is a quaternary oncology service where paediatric patients have been treated since 1929. The service began with 70 children between 1929 and 1944 and 350 between 1945 and 1985.^19^, ^20^ On average, 60 paediatric patients were treated each year at the RBWH until 2014, when paediatric radiation oncology services were centralised at Children's Health Queensland. RTs at the RBWH have applied behavioural therapy techniques to their work with paediatric patients since 1970, aligning with the early era of formal ‘play therapy’ research.^10^, ^21^ In 2005, the RBWH Paediatric Radiation Therapy Group (PRTG) was established, formalising a multidisciplinary care network to support paediatric patients accessing RT services. Members of this group included RTs, Radiation Oncologists (RO), Registered Nurses (RN), Occupational Therapists (OT) and tumour stream coordinators from Cancer Care Services RBWH and the Royal Children's Hospital (RCH). OTs and RTs fulfil a similar role to CCLCs in some RT departments internationally including Australia.^15^ The PRTG aimed to (a) consolidate treatment preparation through the application of behaviour therapy techniques; (b) formally identify staff motivated to provide care to children; (c) coordinate the optimal transfer of care between the paediatric and adult hospital services. Behavioural therapy techniques were incorporated into all RT appointments, and if required, applied during additionally booked play appointments within the RT department. Paediatric patients were offered these play appointments by the RO, the RT care co‐ordinator, or members of the PRTG and could be referred prior to CT planning, after CT planning, during treatment or any combination of these. Patients could utilise as many play appointments as they required to become comfortable with treatment or cease this behavioural intervention based on the family and patient's care requirements.

This study aims to evaluate the use and efficacy of behavioural interventions in the treatment of paediatric oncology patients within the RBWH RT department. It is hypothesised that trends in GA use and play appointment utilisation will show patterns based mainly on the patient's age and therefore linked to cognitive development.

## Methods

This retrospective cross‐sectional study reviewed the GA utilisation for paediatric patients who required RT treatment at the RBWH between 1 January 2010 and 30 June 2014. Using demographic data from the MOSAIQ® radiation oncology information system (Elekta, Stockholm, Sweden), eligible patients were those identified as younger than 18 years of age at the time of referral. Patients referred to our service but not treated were excluded from this review. Project approval was granted by the Children's Health Queensland Hospital and Health Service Human Research Ethics Committee which deemed this quality improvement review exempt from the full human research ethical approval process.

Information extracted included the participant's age at CT planning, gender, treatment course number, diagnosis, technique prescribed, number of treatments prescribed, technique time including the time required to set up and deliver the RT treatment, stabilisation devices, number of additionally booked play appointments and GA status at each encounter. Participants who began treatment with GA but who did not require GA for the whole of their treatment were categorised in the ‘Partial GA’ anaesthetic group, whereas those who used GA for all RT treatments were categorised in the ‘Full GA’ anaesthetic group.

### Statistical methods

Associations between categorical variables and the defined anaesthetic groups (Full GA, Partial GA and No GA) were evaluated using Pearson's chi‐square test, and when the number of cells with an expected count of <5 was >20%, Fisher's exact test was used for evaluation. *P* values of <0.05 were deemed to indicate statistical significance. Logistic regression was performed by looking at the association of age and technique time with the defined anaesthetic groups. Further adjustment for Total Body Irradiation (TBI), an important covariate indicated by the literature, was unable to be performed due to complete separation (when the predictor/s perfectly predict the outcome resulting in invalid estimates). Patients 9 years or older were excluded from the logistic regression due to little or no variability in this age group. The Statistical Package for Social Sciences (SPSS) version 23 was used for analysis.

## Results

Two‐hundred and eighty‐one paediatric patients were referred for RT from 1 January 2010 to until 30 June 2014. One patient was 18 years old, and 6 patients did not proceed to a CT planning appointment after booking, so were excluded from this review. This resulted in 274 participants being included in the review. Five thousand and four hundred and two occasions of service were delivered to the 274 participants, where 1361 (25.2%) were delivered with GA. Seventeen of these participants were not receiving their first course of RT so were excluded from the analysis. The characteristics of the 257 participants treated at the RBWH during the review period are presented in Table 1. The median age of participants was 8 years old, with female patients accounting for less than half (45.9%) of the cohort. The number of patients in each of the diagnosis categories is similar to the population incidence data from U.S. studies.^22^

**Table 1:** Baseline characteristics.

Characteristics	*N* = 257
Age at RT, year, median (IQR)	8 (4–13)
Female, *n* (%)	118 (45.9)
Total RT fractions per course, median (IQR)	20 (7–30)
Diagnosis, *n* (%)	
Brain and CNS tumours	68 (26.5)
Leukaemia	61 (23.7)
Lymphoma	20 (7.8)
Rhabdomyosarcoma	26 (10.1)
Neuroblastoma	22 (8.6)
Wilms tumour	25 (9.7)
Benign hematologic condition	5 (1.9)
Other*	30 (11.7)
Treatment delivery technique, *n* (%)	
3D Conformal Radiation Therapy (3DCRT)	150 (58.4)
Tomotherapy (Tomo)	72 (28.0)
Total Body Irradiation (TBI)	35 (13.6)
Technique time, min, median (range)	15 (10–20)
Stabilisation devices used, *n* (%), (*n* = 222)	117 (52.5)
Treatment commenced (year), *n* (%)	
2010	49 (19.1)
2011	63 (24.5)
2012	57 (22.2)
2013	58 (22.6)
2014^†^	30 (11.7)

CNS, central nervous system; IQR, interquartile range; RT, radiation therapy.

* Connective Tissue Sarcomas, Other Soft Tissue tumours, Epithelial tumours.

^†^ Study was closed in July 2014.

**Figure 1:**
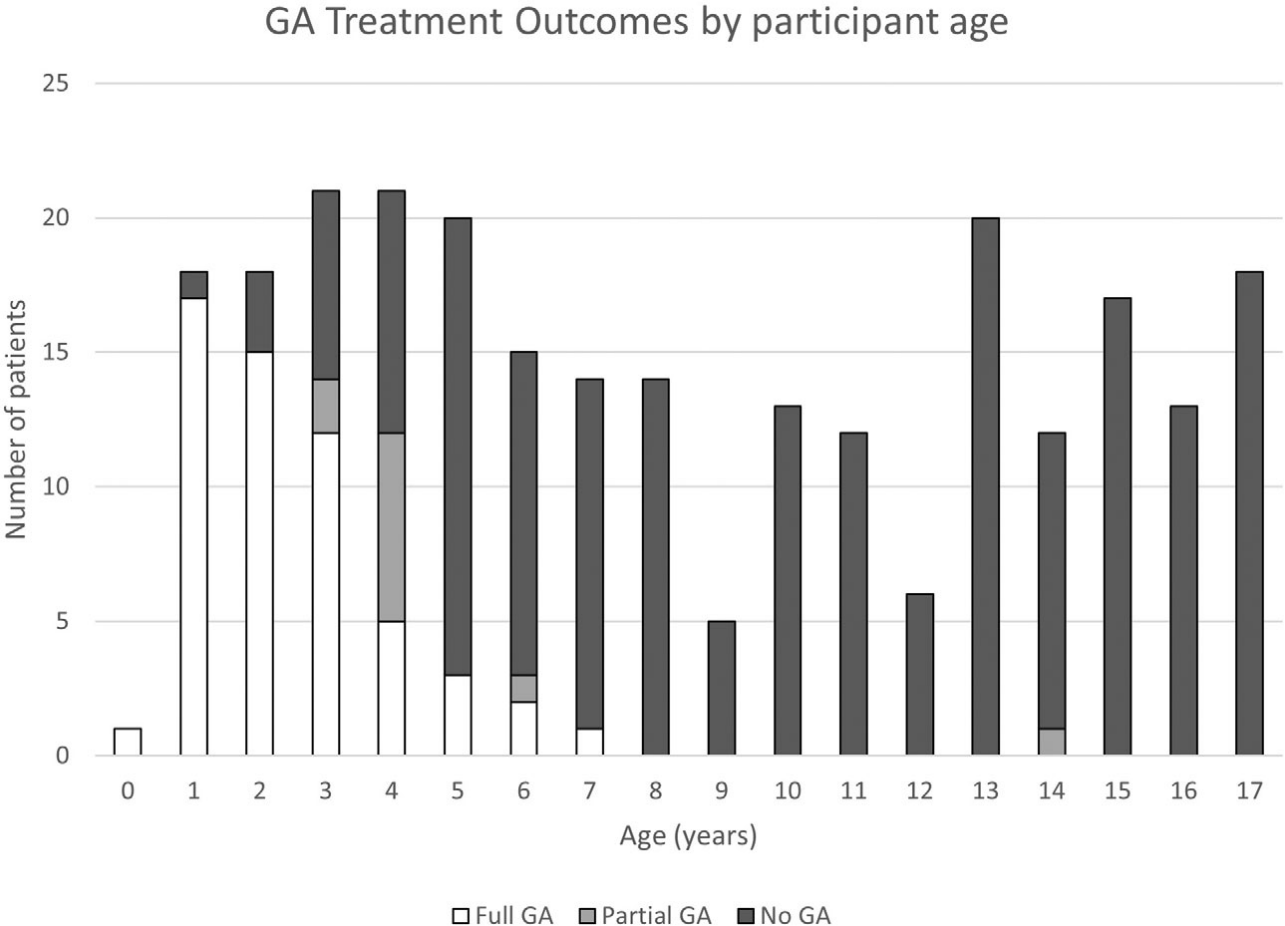
Number of patients and use of GA across all patients, by age, from January 2010 to June 30, 2014.

Sixty‐six of the 257 participants (25.7%) received GA. The use of GA by participant age is shown in Figure 1. The results demonstrate trends in GA utilisation, with the younger participants requiring Full GA or Partial GA more than their older counterparts. The participants who required GA for their entire treatment reduced for each year of increase in age from ages 0 through to 8 years (odds ratio 0.37, 95% confidence interval 0.27–0.51, *P* < 0.001). GA was used for one 14‐year‐olds treated for a central nervous system (CNS) germ cell tumour with full CNS RT delivered on Tomotherapy, whose health deteriorated during treatment. Results did not vary based on a statistical model adjustment for technique time as a covariate.

**Figure 2:**
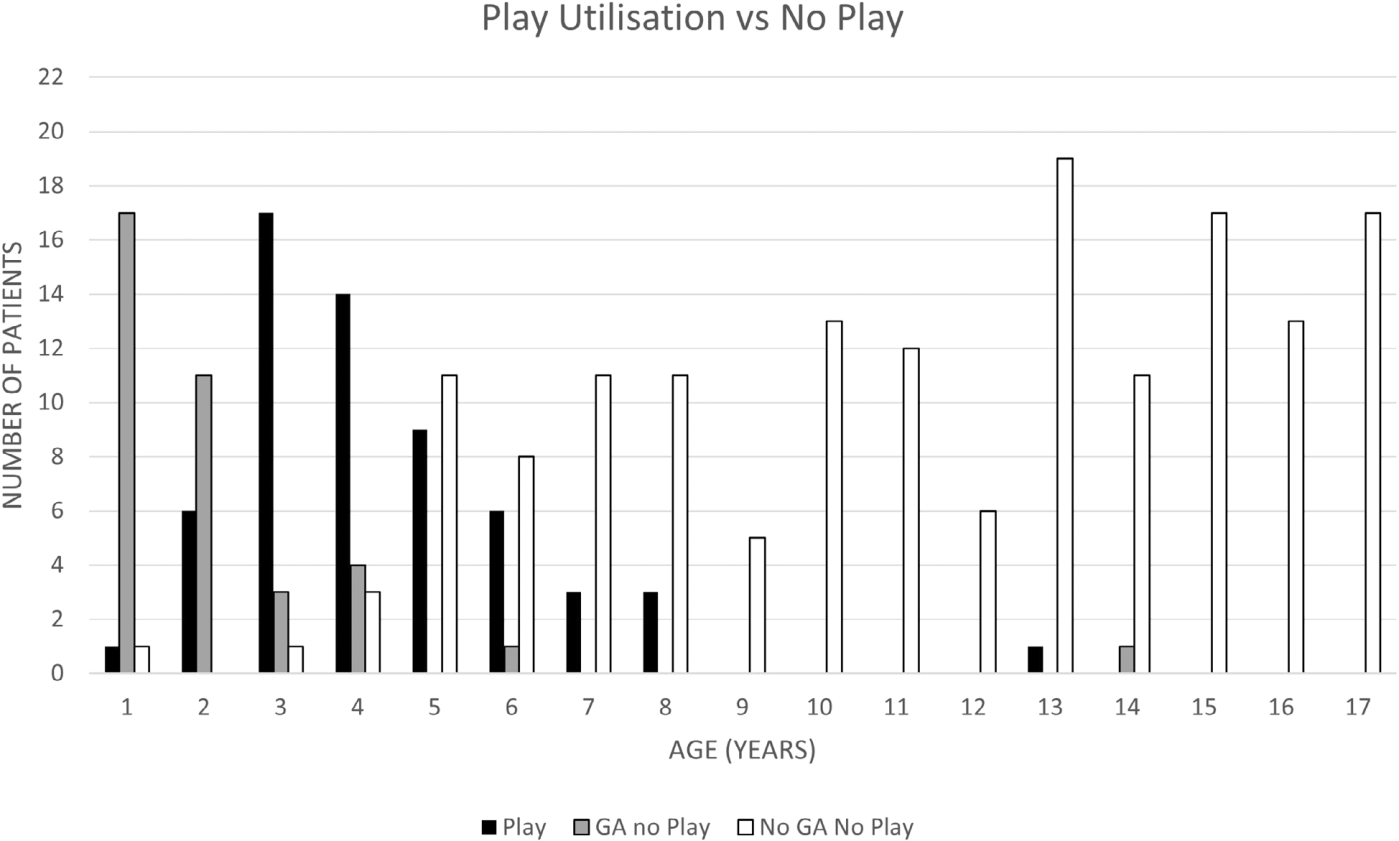
Play appointment utilisation versus no play at the RBWH from January 2010 to June 30, 2014. The table includes the two treatment outcomes of GA and No GA associated with no play.

One hundred and eighty‐one play appointments were booked during the 4.5‐year study period for 60 out of the 257 participants (23%). Participants attended 75% of these bookings (136/181), equating to approximately 30 hours annually of additional clinical load for the RT department. Play appointments were booked but were not attended in some cases due to a decision to proceed with Full GA or because the participant could already comply with treatment requirements and no longer required GA. The use of play appointments by age is demonstrated in Figure 2.

Play appointments were increasingly used for participants aged 2 and 3 years and gradually decreased to 9 years. Thirty‐five per cent of 2‐year‐olds used play appointments (6/17) compared to 81% of 3‐year‐olds (17/21) (Fig. 2). Participants older than 3 years were increasingly able to comply with the requirements of RT without play appointments (No GA No play). Children 9 years and older generally did not receive GA for treatment and did not utilise play appointments (Fig. 2). One 13‐year‐old treated for an astrocytoma with cranial RT utilised one play appointment to assist them in meeting the requirements of treatment in a mask, and the one 14‐year‐olds previously mentioned required GA (Fig. 2). Play appointments were most utilised in 3–8‐year‐olds, booked for 52 of 106 patients (49%). For these 52 participants, the median age was 4 years (IQR 3–5) and the median number of play appointments booked was 2 (IQR 1.5–4). This result prompted a review of the demographics of the 3–8‐year‐old cohorts.

The relationships between receiving anaesthesia and patient and treatment characteristics in 3–8‐year‐olds are outlined in Table 2. Variables including gender, the use of mask stabilisation devices over patients' faces, and the daily time required for the treatment technique were not found to be significantly associated with anaesthesia use among this group (*P* = 0.22, *P* = 0.21 and *P* = 0.97) (Table 2).

**Table 2:** Factors associated with anaesthesia (3–8 years).

Variable	Categories	Anaesthesia			*P* value
		No GA	Partial GA	GA	
Gender	Male	36 (62.1%)	6 (10.3%)	16 (27.6%)	0.22
	Female	37 (77.1%)	4 (8.3%)	7 (14.6%)	
Stabilisation	No	33 (73.3%)	5 (11.1%)	7 (15.6%)	0.21
	Yes	31 (59.6%)	5 (9.6%)	16 (30.8%)	
Technique time	<15 min	21 (67.7%)	3 (9.7%)	7 (22.6%)	0.97
	15+ min	43 (65.2%)	7 (10.6%)	16 (24.2%)	

**Figure 3:**
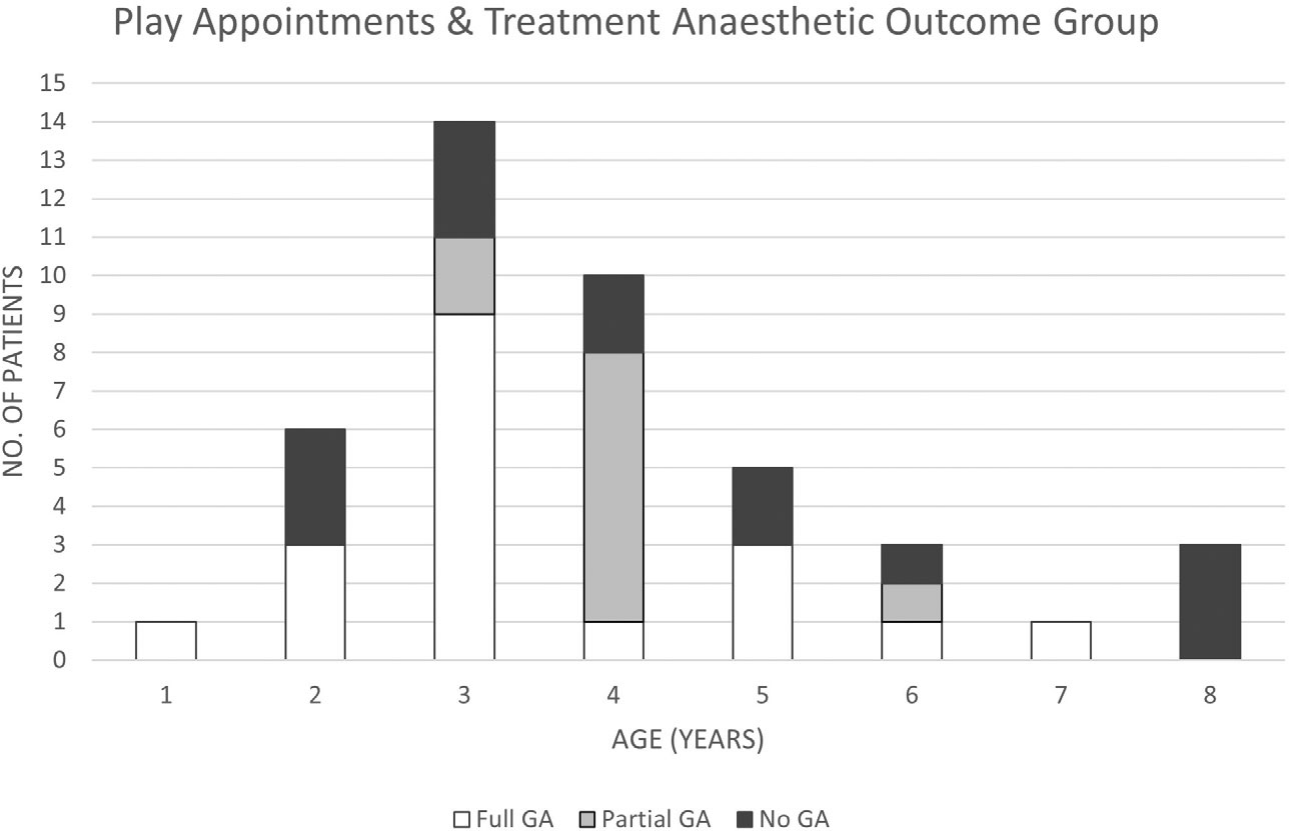
Play appointments and Treatment anaesthetic outcome groups per age—1–8 years.

**Figure 4:**
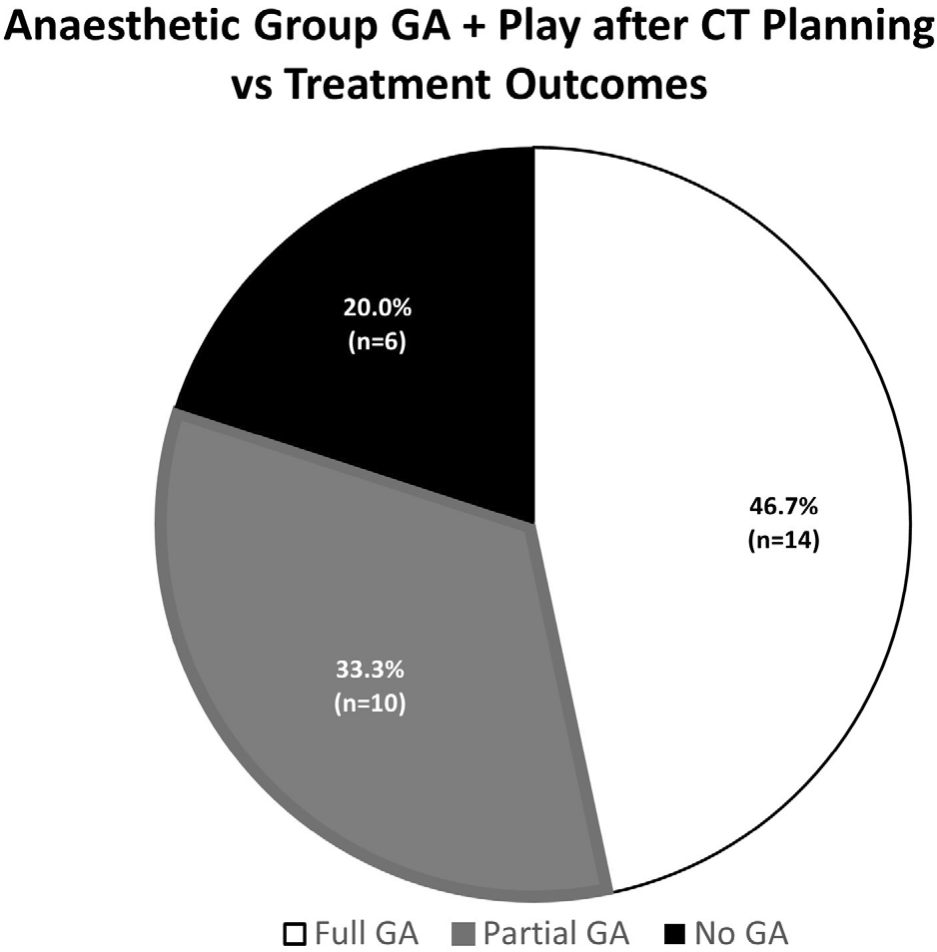
Three to eight‐year‐old patients’ treatment outcome when they were categorised as GA plus Play after CT Planning, from January 2010 to June 30, 2014.

Seventy‐eight per cent (83/106) of 3–8‐year‐old patients had the treatment outcome of ‘No GA’ or ‘Partial GA’, whereas 22% (23/106) used GA for their full treatment course (Fig. 1). When the treatment outcomes of the play categories were further reviewed by age, the data displayed more participants in the ‘Partial GA’ and ‘No GA’ treatment outcome groups than ‘Full GA’ in 4–8‐year‐olds (*n* = 16/22, 72.7%) (Fig. 3). Children 3 years and younger who used play appointments were more likely to continue to use GA, with a treatment outcome of full GA in 61.9% (13/21) of 1–3‐year‐olds (Fig. 3). Encouragingly, 33.3% (10/30) of 3–8‐year‐olds who were categorised as full GA for treatment and received play appointments were able to cease GA, resulting in a ‘Partial GA’ treatment outcome, while 20% (6/30) proceeded to treatment with no GA. Combining these treatment outcome groups demonstrates that 53.3% of 3–8‐year‐olds who were categorised as full GA changed their treatment anaesthetic outcome with the addition of play appointments (Fig. 4).

When the technique of TBI was independently evaluated, none of the 34 patients who required this treatment required GA for CT planning or treatment. This subgroup of patients ranged in age from 3 years to 17 years with a median age of 7 years (IQR 3–13).

## Discussion

This study aimed to evaluate the use and efficacy of behavioural interventions in the treatment of paediatric oncology patients within our RT department. Behavioural therapy resources in the form of play appointments were predominately utilised in 3–8‐year‐old patients regardless of diagnosis or treatment techniques applied. Variables specific to the patient and their health concerns, other than age, were not significantly associated with anaesthesia use. As anticipated, younger patients required full or partial GA more than their older counterparts, aligning with the study's hypothesis. GA utilisation was reported at 25.7% over the study period, similar to other international RT centres that integrated behavioural therapy practises into their departments (Table 3).^6^, ^16^, ^23^ Children 9 years and older generally did not receive GA for treatment (Fig. 1) and did not utilise play appointments (Fig. 2), providing evidence that above 9 years, patients do not generally require play appointments to comply with RT requirements. This statement does not hold true when paediatric patients experience significant health declines affecting their cognition during RT. Children 3 years and younger who used play appointments were more likely to continue to require GA in comparison to children aged 4–8 years (Fig. 3). Results showed that 53.3% of patients who were categorised as full GA after CT planning and received play appointments were able to cease GA either before treatment (No GA 6/30) or during treatment (Partial GA 10/30). Therefore, a portion of 3–8‐year‐olds did not require Full GA with the addition of play appointments (Fig. 4). Services could consider targeting behavioural therapy resources such as play appointments for 3–8‐year‐old cohorts. These results support the adoption of behavioural therapy interventions in the treatment of paediatric oncology patients, with benefits to the child, carers and health service providers.^2^, ^6^, ^7^, ^9^

**Table 3:** Comparison of anaesthesia use per patient in published Radiotherapy literature, matched by age range.

Publication	Population	Novel Intervention applied	Study size, *n*	Age range, year	GA %	RBWH review % GA as per the population and age range in the published literature
Ntoukas et al^18^	All		779	0 to 19	12.8*	25.7 (0–18 years)
Hinikler et al^23^	All	Audio‐visual	25	3 to 12	36.0	23.2
Scott et al^16^	All		304	3 to 12	40.8	23.2
		CCLS	118	5 to 8	28.8	9.4
Grissom et al^17^	CNS	CCLS	116	5 to 12	47.4	13.0
Willis et al^24^	All	Audio‐visual	37	2 to 6	73.0	48.9
Haeberli et al^25^	All		90	<1.6–19	8.9	25.7 (0–18 years)
Scott et al^9^	All	Play therapy	63	2 to 5	10.8	54.4
Seiler et al^6^	All	Nil	193	0 to 14	37.0	31.4
	Leukaemia	Nil	51	0 to 14	39.0	7.4

CCLS, Certified Child Life Specialists; CNS, central nervous system; GA, General Anaesthetic.

* Number based on occasions of service.

Seiler et al^6^ in 2001 analysed anaesthesia use in patients from 0 to 14 years, with 37% of these patients requiring sedation compared to 31.4% of 0–14‐year‐olds in the RBWH cohort. The median age in the Seiler et al^6^ study was considerably lower at 3.8 years in comparison to 6.4 years in the RBWH cohort, impacting the validity of comparing the studies. The Seiler et al^6^ publication is the only noted study not to exclude leukaemia patients from their results, allowing some comparison with our data, as the RBWH was the primary treatment centre in Queensland for this diagnosis and the RT technique of TBI at that time. Anecdotal observations by oncology professionals have suggested that the treatment technique for TBI used at the RBWH and the behavioural therapy work performed by the PRTG may be a contributing factor that resulted in no TBI patients requiring anaesthesia.

There were several limitations in our study. This study did not contain a control group, as the PRTG formally applied behavioural therapy practices in 2005. Prior to 2010, paediatric patients were treated on a dedicated linear accelerator, often receiving access to the bunker for play appointments. In 2010, a remodel of our department resulted in reduced access to this linear accelerator. It is believed that this circumstance would have influenced a portion of the results of this study. Due to this remodel, a new treatment machine was introduced in late 2010. Protocols applied to this machine meant that if any patients were not deemed fully compliant, they were sedated to avoid the possibility of movement during treatment delivery. It is also important to note that some parents were unwilling to deviate from full GA, even when the patient's age and health indicated that they could potentially proceed to receive RT without anaesthesia. PRTG members consistently and comprehensively consulted all parents about behavioural therapy and the benefits of patients not receiving anaesthetic daily. Information regarding the decision to not deviate from full GA despite perceived capacity is anecdotal and was not recorded in any way by the department.

## Conclusion

Behavioural therapy techniques have been widely adopted as a valuable tool in the treatment of paediatric patients within healthcare. Radiation therapy departments could benefit from the adoption of interventions such as behavioural therapy practises and play appointments, to assist in the provision of the optimal model of care for paediatric patients. Children 3 years and younger were shown not as likely to change their GA requirements with the addition of play appointments. Children older than nine were not likely to require play appointments to be compliant. Services could consider targeting behavioural therapy resources such as play appointments for the 3–8‐year‐old cohorts. Encouragingly, over half of 3–8‐year‐old patients, who were categorised as full GA for treatment after CT planning and proceeded to play appointments, did not proceed to a full course of GA attributed to the value of play appointments applied by the PRTG.

## Acknowledgments

We would like to thank Dr Roger Allison for his support of the PRTG, his conceptual support for this publication and his significant contribution to the successful treatment of paediatric patients at the RBWH over his career. We would also like to thank Dr Cathy Hargrave and Allison Dry for their support and review of the manuscript. Finally, we would like to extend a thank you and congratulations to all the RBWH radiation therapy staff who have championed the care of paediatric patients from 1929 to 2014.

## Conflict of interest

The authors declare no conflict of interest.

## Ethics and integrity policy statement

This manuscript was prepared using retrospective de‐identified patient demographic data from the Oncology Information Management system, MOSAIQ®. Project approval was granted by the Children's Health Queensland Hospital and Health Service Human Research Ethics Committee who deemed this a quality improvement review exempt from the full human research ethical approval processes. This manuscript was prepared with no additional funding, utilising in‐kind expenditure within Queensland Health.

## Data Availability

The data that support the findings of this study is available on request from the corresponding author. The data is not publicly available due to privacy or ethical restrictions.
